# Supplementary material for: Insight into Unusual Supramolecular Self-Assemblies of Terthiophenes Directed by Weak Hydrogen Bonding
Source: Int J Mol Sci. 2023 Jul 5;24(13):11127. doi: 10.3390/ijms241311127 (PMC10342644; doi:10.3390/ijms241311127)
Supplement: Supplementary file 1 [file ijms-24-11127-s001.zip › ijms-2474996-supplementary.pdf]

International Journal of *Molecular Sciences*

MDPI

Supplementary Materials

Insight into Unusual Supramolecular Self-Assemblies of Terthiophenes Directed by Weak Hydrogen Bonding

Shiv Kumar [1], Kristof Van Hecke [2] and Franck Meyer [1,*]

[1] Microbiology, Bioorganic and Macromolecular Chemistry (MBMC) Unit, Faculty of Pharmacy, Université Libre de Bruxelles, 1050 Brussels, Belgium; shiv.kumar@ulb.be

[2] XStruct, Department of Chemistry, Ghent University, Krijgslaan 281-S3, 9000 Ghent, Belgium; kristof.vanhecke@ugent.be

* Correspondence: franck.meyer@ulb.be; Tel.: +32-(0)-2-650-51-96

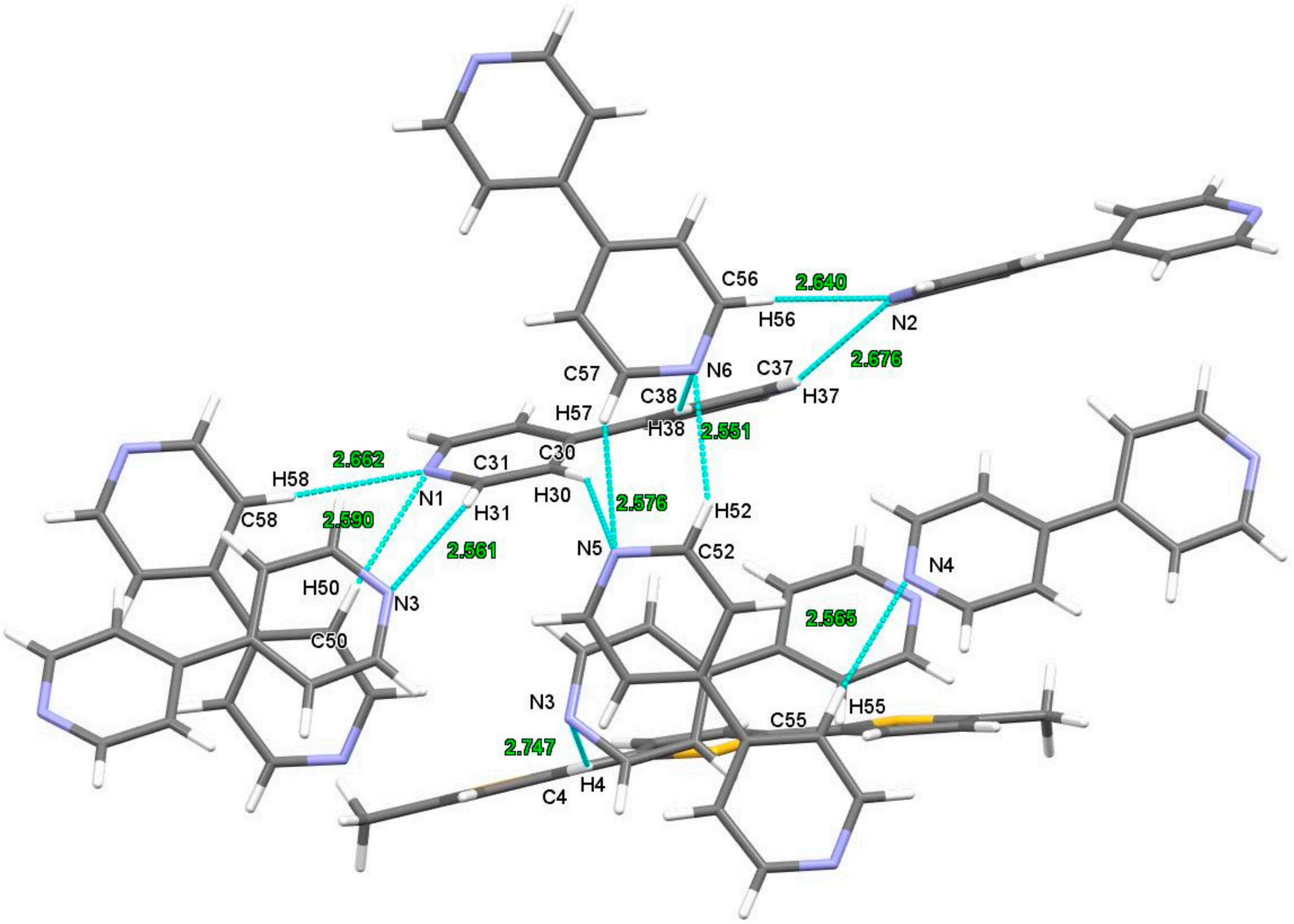

Figure S1. Complex network of HBs involving C-H···N interactions in **DM3T-44BiPy** co-crystal with labeled atoms.

International Journal of *Molecular Sciences*

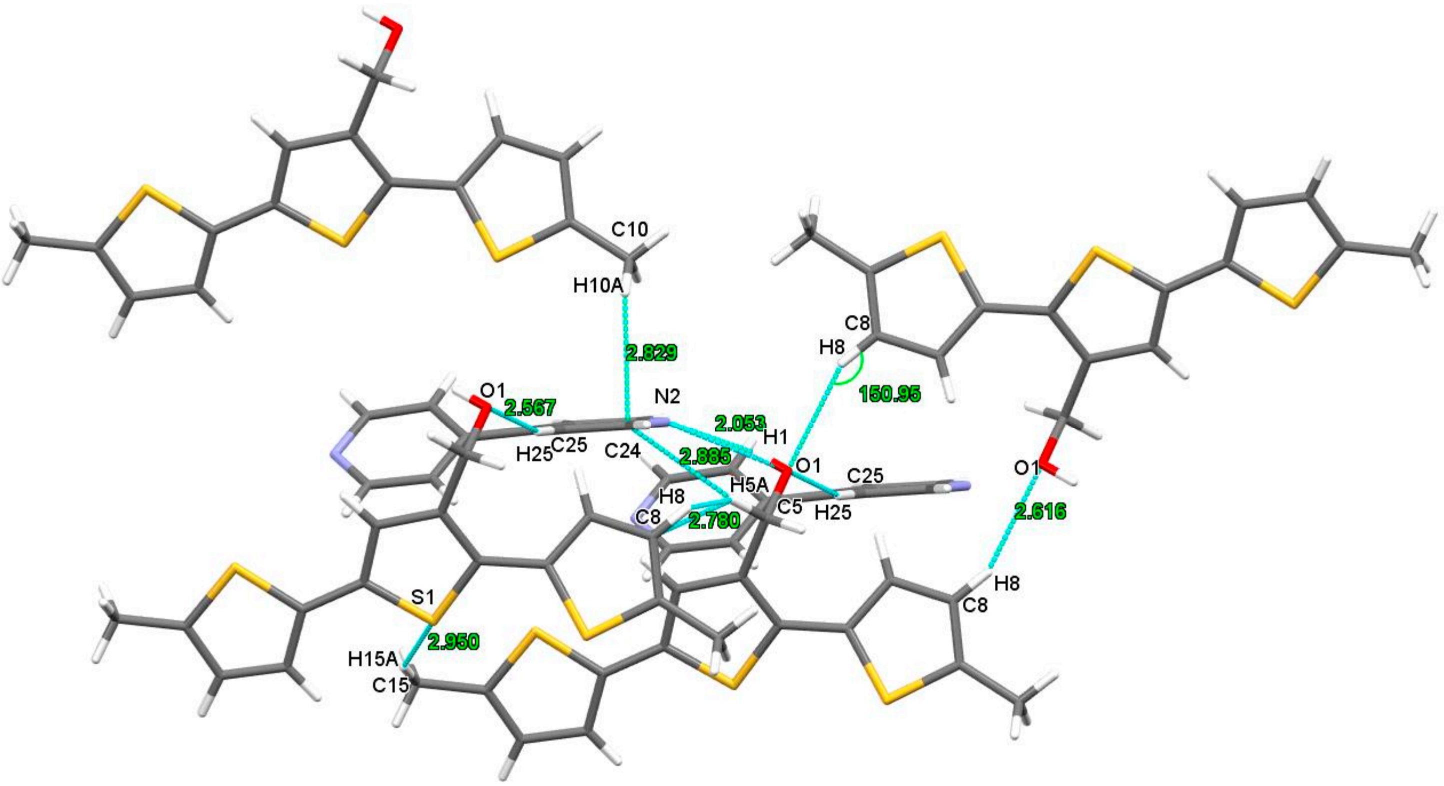

Figure S2. Labeled atoms involved in HBs of **DM3TMeOH-44BiPy** co-crystal.

International Journal of
Molecular Sciences

MDPI

Code	Shape index	Curvedness
**DM3T**		
**DM3T-44BiPy**		
**DM3TMeOH-44BiPy**		

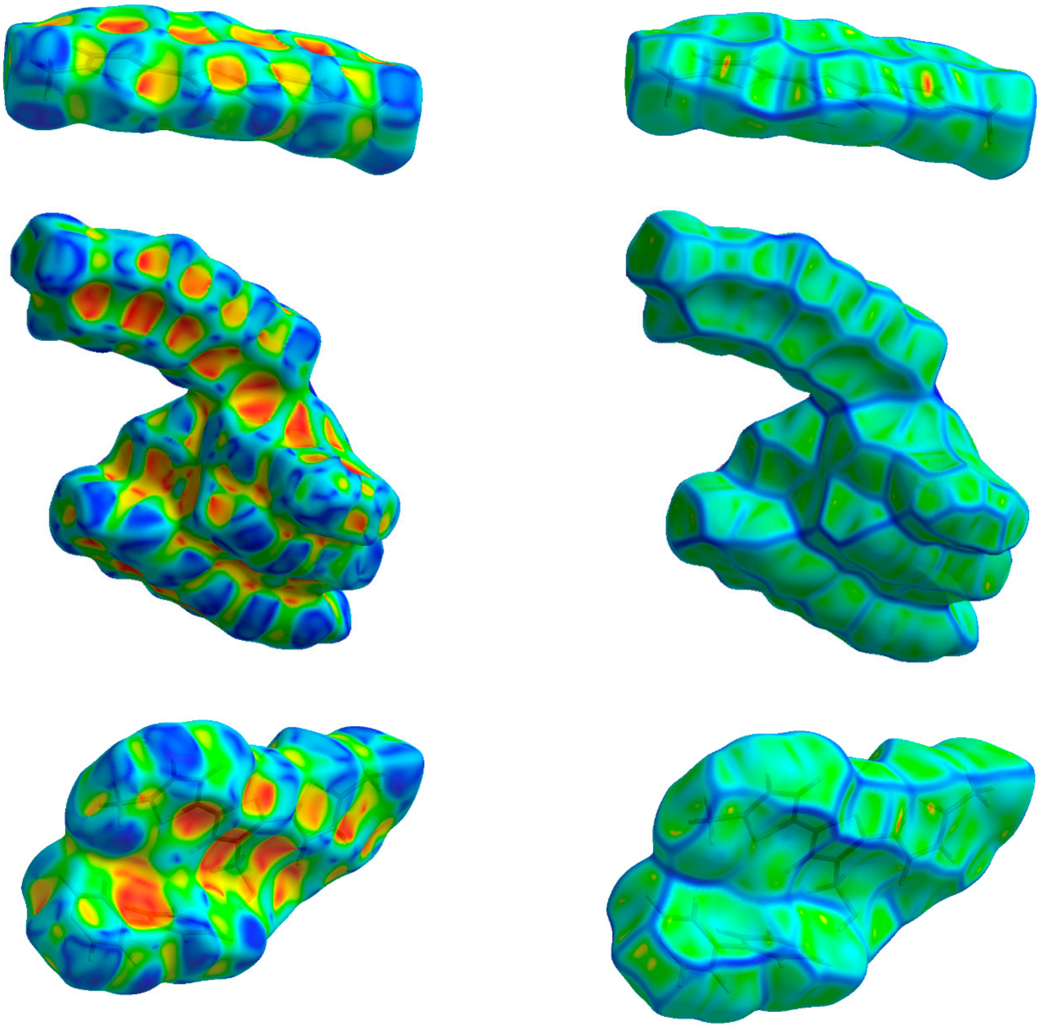

Figure S3. Shaped index and curvedness surfaces of **DM3T, DM3T-44BiPy** and **DM3TMeOH-44BiPy**.

International Journal of
Molecular Sciences

MDPI

Table S1 X-ray crystal data and refinement parameters

Crystal data	**DM3T**	**DM3T-44BiPy**	**DM3TMeOH-44BiPy**
Empirical formula	$C_{14}H_{12}S_3$	$C_{58}H_{48}N_6S_6$	$C_{25}H_{22}N_2OS_3$
Formula weight	276.42	1021.38	462.62
Temperature/K	100(2)	100(2)	100(2)
Crystal system	monoclinic	triclinic	triclinic
Space group	$P2_1$	$P\bar{1}$	$P\bar{1}$
a/Å	11.7994(7)	9.6364(3)	7.40050(10)
b/Å	7.5426(4)	16.3664(5)	9.14680(10)
c/Å	14.9046(7)	16.6876(5)	17.0193(3)
α/°	90	76.936(3)	80.0500(10)
β/°	103.631(6)	82.602(3)	82.8270(10)
γ/°	90	80.237(3)	82.0910(10)
Volume/$Å^3$	1289.12(12)	2515.37(14)	1117.88(3)
Z	4	2	2
ρ_{calc}g/cm^3	1.424	1.349	1.374
μ/mm^{-1}	0.548	2.871	3.189
F(000)	576.0	1068.0	484.0
Crystal size/mm^3	0.58 × 0.16 × 0.04	0.23 × 0.12 × 0.09	0.27 × 0.21 × 0.15
Radiation	Mo Kα (λ = 0.71073 Å)	Cu Kα (λ = 1.54184 Å)	Cu Kα (λ = 1.54184 Å)
2Θ range for data collection/°	5.024 to 50.034	5.46 to 148.44	5.3 to 147.546
Index ranges	-14 ≤ h ≤ 14, -8 ≤ k ≤ 8, -17 ≤ l ≤ 17	-11 ≤ h ≤ 11, -20 ≤ k ≤ 20, -20 ≤ l ≤ 19	-8 ≤ h ≤ 9, -11 ≤ k ≤ 11, -20 ≤ l ≤ 20
Reflections collected	62559	42640	39576
Independent reflections	4531 [R_{int} = 0.1053, R_{sigma} = 0.0460]	10007 [R_{int} = 0.1028, R_{sigma} = 0.0858]	4440 [R_{int} = 0.0323, R_{sigma} = 0.0185]
Data/restraints/parameters	4531/247/312	10007/0/635	4440/0/285
Goodness-of-fit on F^2	1.005	0.991	1.045
Final R indexes [I>=2σ (I)]	R_1 = 0.0679, wR_2 = 0.1261	R_1 = 0.0501, wR_2 = 0.1039	R_1 = 0.0311, wR_2 = 0.0782
Final R indexes [all data]	R_1 = 0.0901, wR_2 = 0.1371	R_1 = 0.0881, wR_2 = 0.1210	R_1 = 0.0369, wR_2 = 0.0820
Largest diff. peak/hole / e $Å^{-3}$	1.41/-0.56	0.48/-0.29	0.32/-0.28
CCDC Number	2266731	2266732	2266733

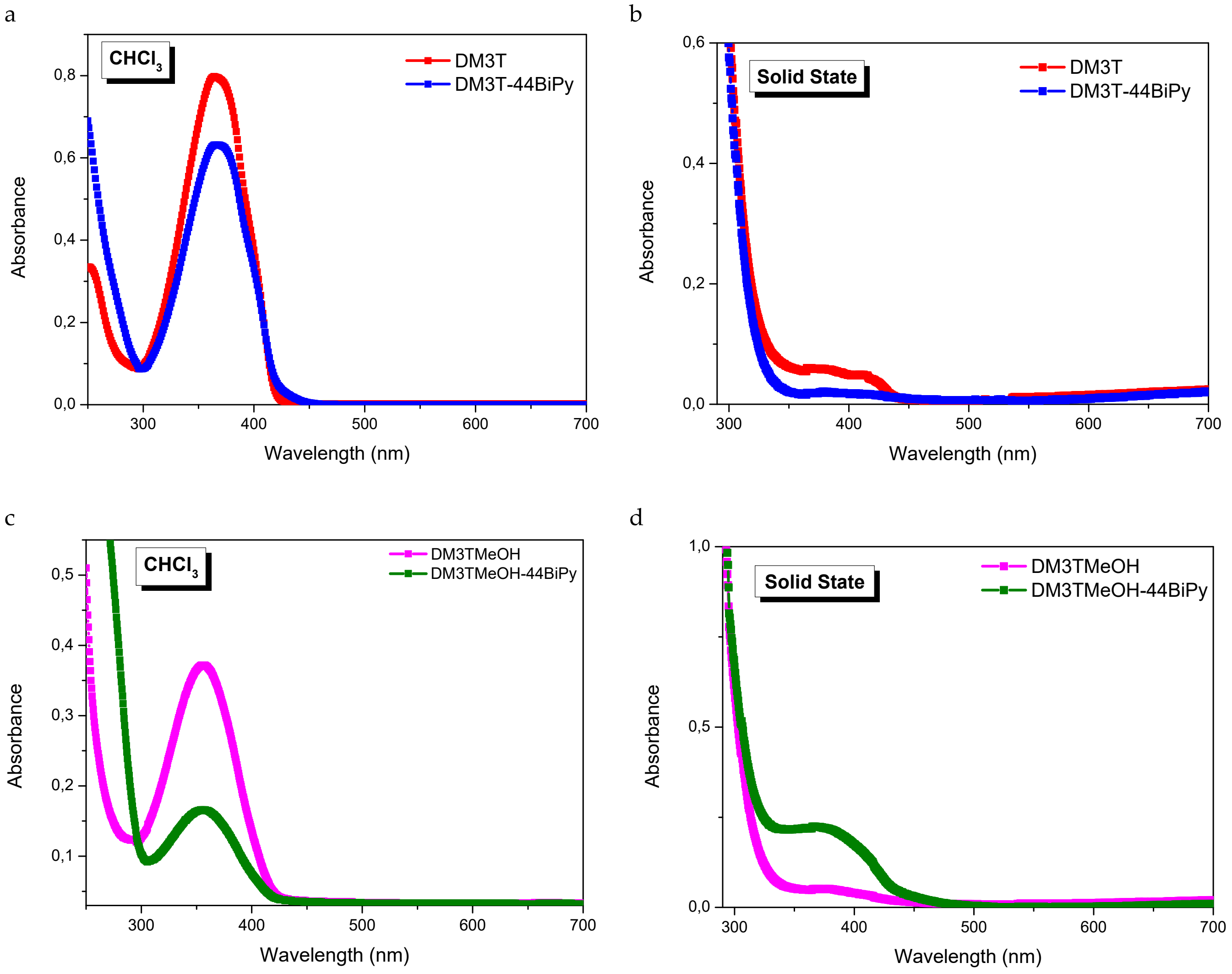

Figure S4. UV-visible absorption spectrum of **DM3T**, **DM3T-44BiPy, DM3TMeOH,** and **DM3TMeOH-44BiPy** recorded in $CHCl_3$ solution (a, c) and solid-sate (b, d) at room temperature.

International Journal of
Molecular Sciences

MDPI

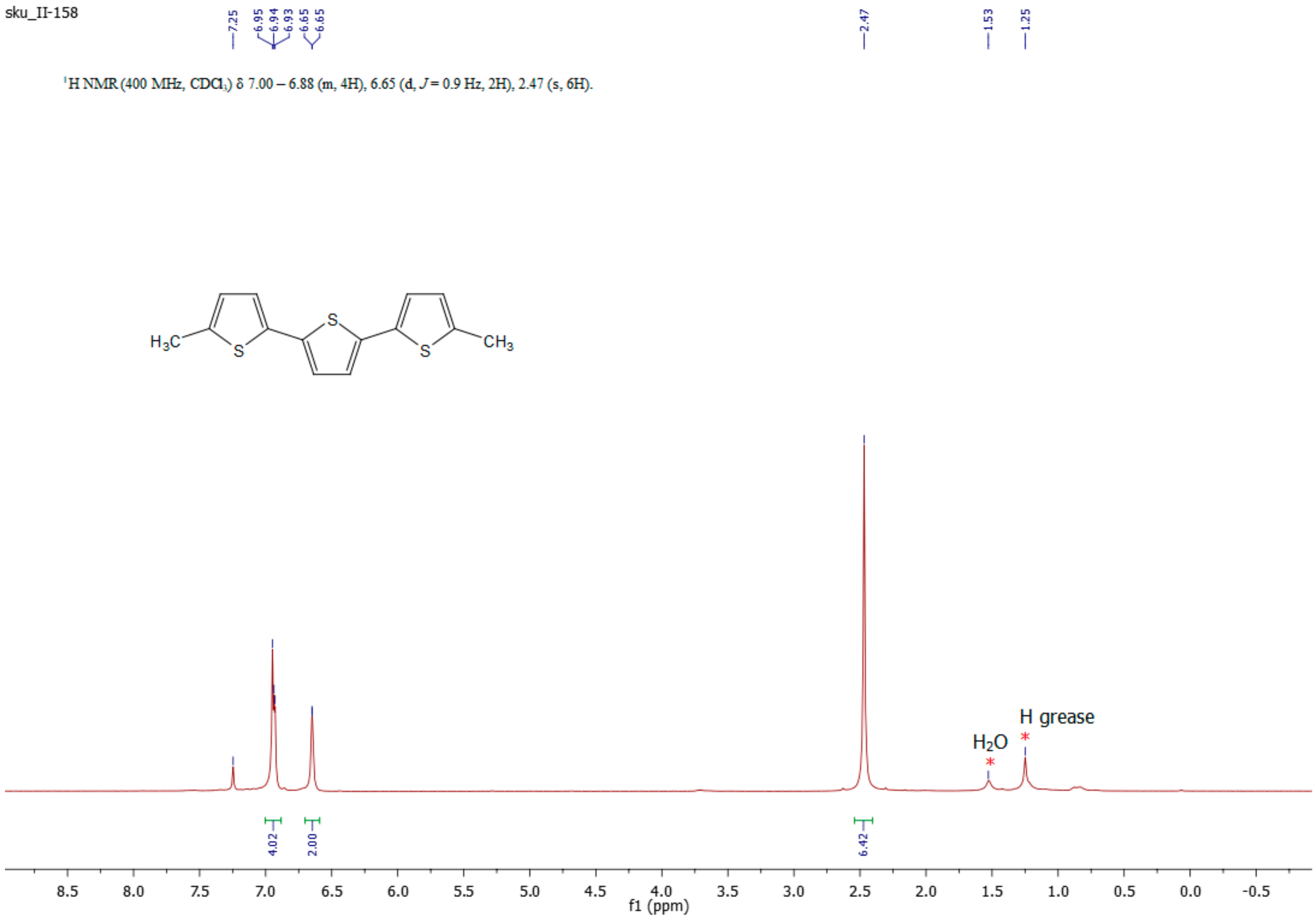

Figure S5. ^{1}H NMR of **DM3T** in $CDCl_3$.

International Journal of
Molecular Sciences

sku_II-158
13C BBD

^{13}C NMR (101 MHz, $CDCl_3$) δ 139.25, 136.13, 135.00, 126.04, 123.58, 123.49, 15.44.

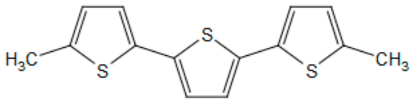

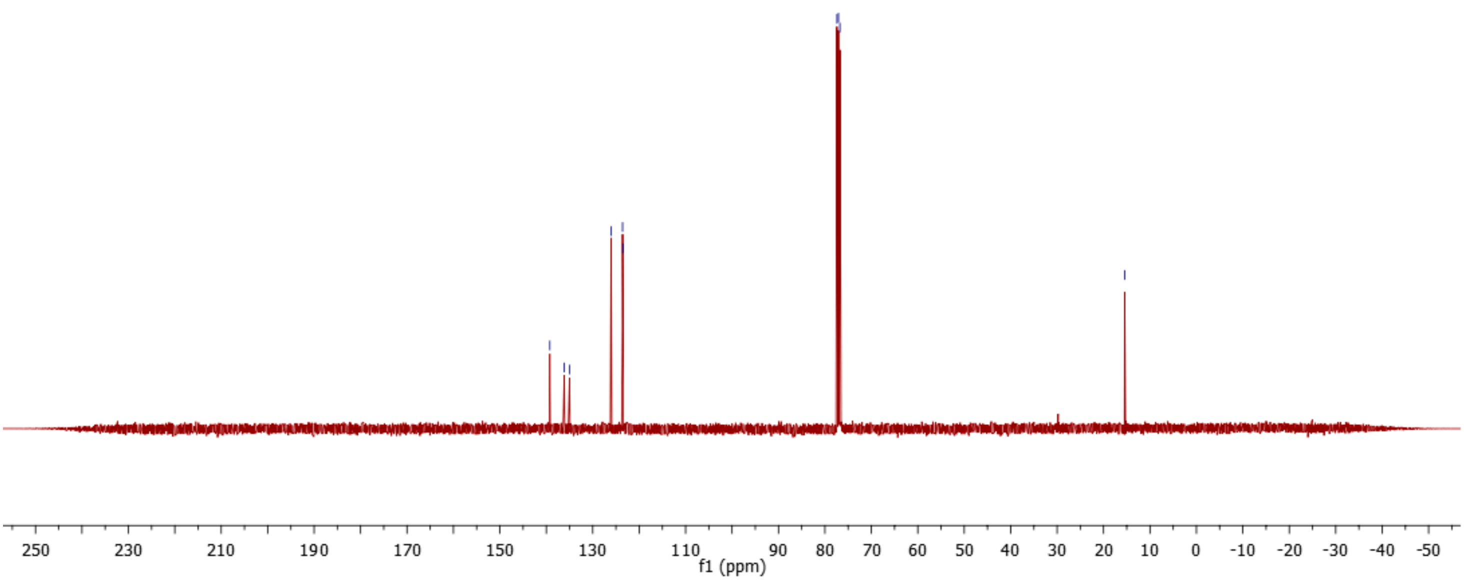

Figure S6. ^{13}C NMR of **DM3T** in $CDCl_3$.

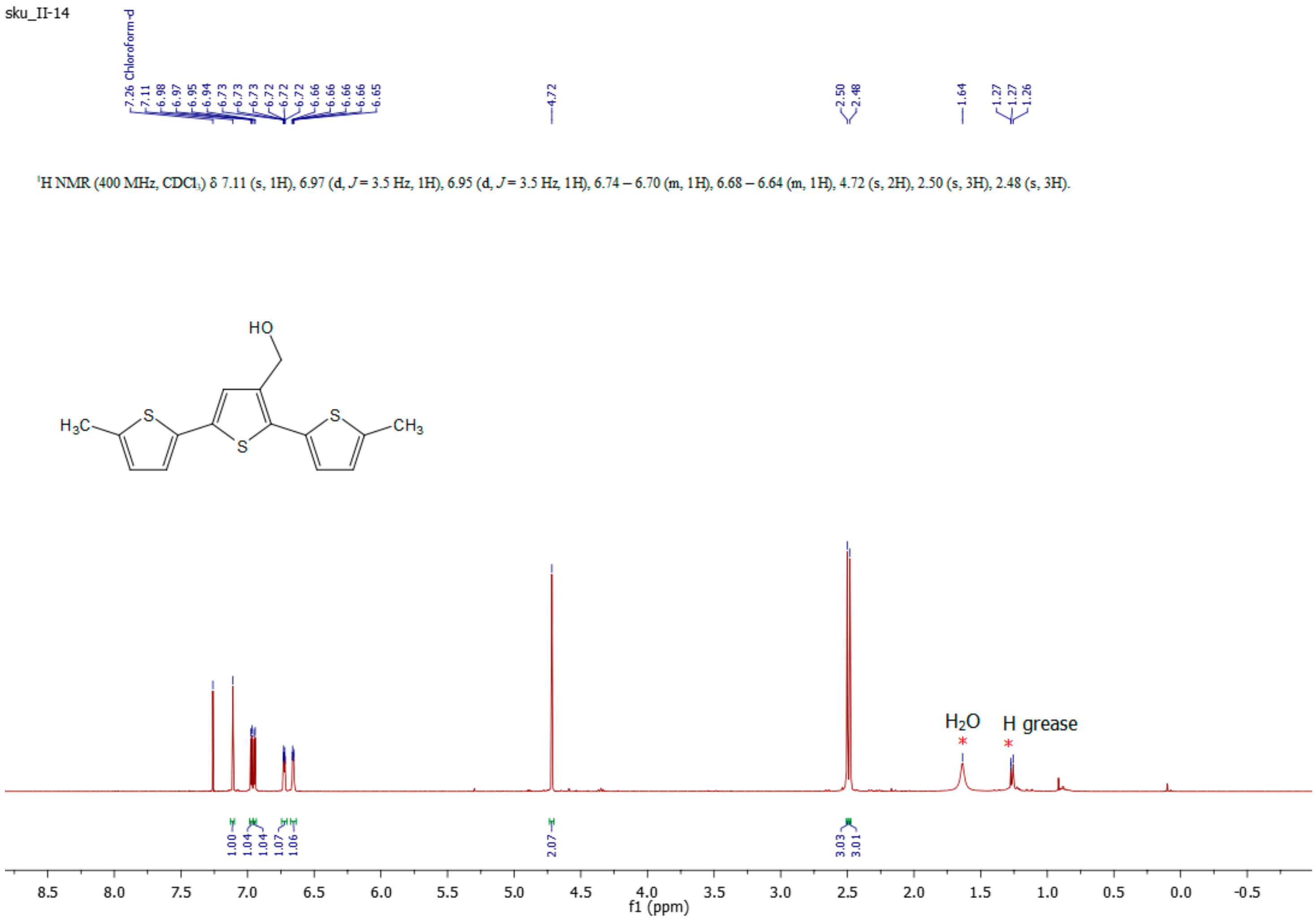

Figure S7. ^{1}H NMR of **DM3TMeOH** in $CDCl_3$.

International Journal of *Molecular Sciences*

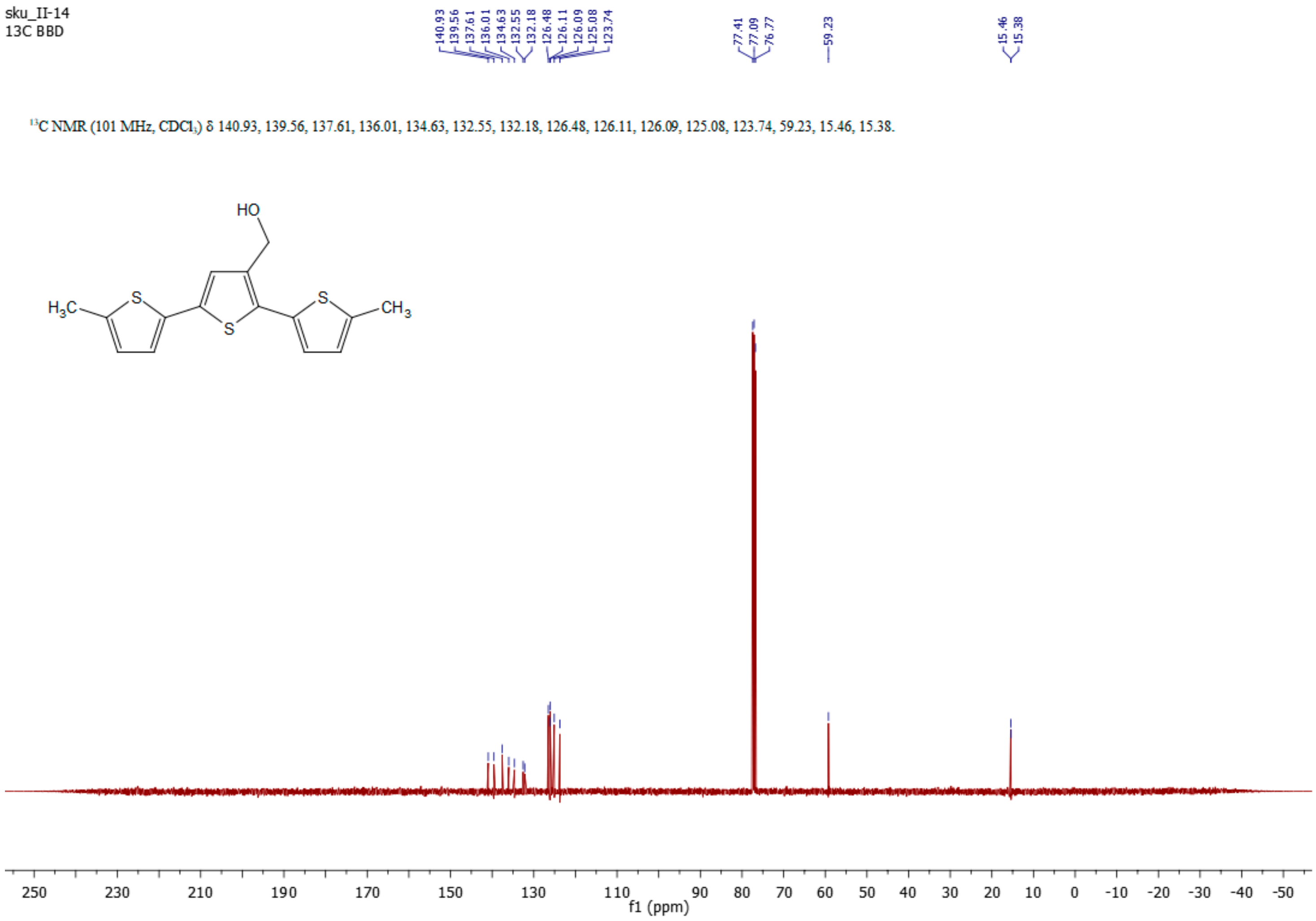

Figure S8. ^{13}C NMR of **DM3TMeOH** in $CDCl_3$.
